# Outcomes of intramedullary nailing for acute proximal humerus fractures: a systematic review

**DOI:** 10.1007/s10195-015-0384-5

**Published:** 2015-10-27

**Authors:** Jason Wong, Jared M. Newman, Konrad I. Gruson

**Affiliations:** Department of Orthopaedic Surgery, Albert Einstein College of Medicine, 1250 Waters Place, 11th Floor, Bronx, NY 10461 USA

**Keywords:** Systematic review, Intramedullary nail, Proximal humerus fracture, Outcomes

## Abstract

**Background:**

While proximal humerus fractures remain common within the elderly population, the optimal treatment method remains controversial. Intramedullary nailing has been advocated as an effective and less invasive surgical technique. The purpose of this study is to elucidate the demographics, outcomes, and complications of intramedullary nailing for acute, displaced proximal humerus fractures.

**Materials and methods:**

Multiple computerized literature databases were used to perform a systematic review of English-language literature. Studies that met our stated criteria were further assessed for the requisite data, and when possible, similar outcome data were combined to generate frequency-weighted means.

**Results:**

Fourteen studies with 448 patients met our inclusion criteria. The frequency-weighted mean age was 64.3 years, and mean follow-up was 22.6 months. Females accounted for 71 % of the included patients. Three-part fractures (51 %) were most commonly treated. The overall frequency-weighted mean Constant score was 72.8, and American Shoulder and Elbow Surgeons (ASES) score was 84.3. Frequency-weighted mean forward elevation, abduction, extension, and external rotation were 137.3°, 138.4°, 33.8°, and 43.1°, respectively. The Constant score for two- and three-part fractures was significantly higher than for four-part fractures (*p* = 0.007 and *p* = 0.0009, respectively). The reoperation rate for two-, three-, and four-part fractures was 13.6, 17.4, and 63.2 %, respectively.

**Conclusions:**

Intramedullary nailing of acute, displaced two- and three-part proximal humerus fractures yields satisfactory clinical outcomes, although reoperation and complication rates remain high. Use of this implant for four-part fractures cannot be recommended until further clinical studies with larger patient numbers are available.

**Level of evidence:**

Level IV, Systematic review.

## Introduction

Proximal humerus fractures account for 4–5 % of all fractures and occur most frequently in elderly female patients [1–4]. From 1999 to 2005 there was a 25 % relative increase of proximal humerus fractures treated surgically [2]. Commonly utilized techniques include percutaneous fixation [5], open reduction with locking plate fixation (ORIF) [6], intramedullary nailing (IMN) [7, 8], hemiarthroplasty (HA) [9], and reverse shoulder arthroplasty (RSA) [10]. Continued debate exists as to which of these represents the “gold standard” to manage acute, displaced proximal humerus fractures. Amongst nonarthroplasty techniques, some studies have reported on successful use of locked plating in treating the more complex, three- and four-part fractures [6]. Others have shown similarly good clinical outcomes with the use of a locked, antegrade intramedullary nail [11, 12]. The purported advantages of IMN include decreased soft tissue disruption, preservation of blood supply, and shorter operative time.

The purpose of this study is to critically evaluate the outcomes following locked, antegrade IMN of acute, displaced proximal humerus fractures reported in the literature and present a concise systematic review. Specifically, we attempt to determine: (1) the demographics of patients who undergo IMN for two-, three-, and four-part proximal humerus fractures; (2) the outcomes following IMN for acute proximal humerus fractures, including functional scores and range-of-motion (ROM) data; (3) the rate and types of complications following IMN for displaced proximal humerus fractures; and (4) any difference in outcomes between two-, three-, and four-part proximal humerus fractures.

## Methods and materials

We used the PubMed, EMBASE, ScienceDirect, and Web of Science computerized literature databases to search all years from the beginning of the database through April 2014. Articles were retrieved by using the following keywords: “intramedullary nailing proximal humerus,” “intramedullary nailing proximal humerus fracture,” and “proximal humeral nailing.” In addition to these keywords, we utilized the medical subject heading (MeSH) “shoulder fractures” combined with “fracture fixation, intramedullary” to maximize search specificity and sensitivity in the PubMed database.

Inclusion criteria for studies in this systematic review included published studies that: (1) were written in the English language; (2) had a minimum clinical follow-up of 12 months; (3) reported on the use of antegrade IMN for acute two-, three-, and four-part proximal humerus fractures; (4) utilized at least one validated outcome measure; and (5) had ≥10 patients for review. Exclusion criteria included studies that: (1) were review articles, case reports, or technical papers; (2) provided combined outcomes data for fracture-dislocations and/or proximal humeral fractures with diaphyseal extension without individual data for acute fractures; (3) involved the use of flexible intramedullary devices; (4) involved fractures resulting from bony metastasis; and (6) did not explicitly report a minimum 12-month follow-up. Finally, the reference lists of all the full-text papers were reviewed to identify any additional studies that met the stated inclusion criteria.

The search strategy was independently implemented by two of the authors to select references from the above-mentioned databases. Disagreement between two independent reviewers was resolved by consensus and arbitration of the senior author. The article titles and abstracts were screened according to the eligibility criteria. The full texts of the articles that met the inclusion criteria were thoroughly reviewed. The following data were extracted from the articles: (1) number of acute, displaced proximal humerus fractures treated with IMN, including the number of two-, three-, and four-part fractures; (2) mean patient age; (3) mean, minimum, and range of follow-up; (4) patient gender; (5) use of a surgical or nonoperative control group; (6) mean time from injury to surgery; (7) functional outcome scores; and (5) complications and reoperation rates.

**Fig. 1:**
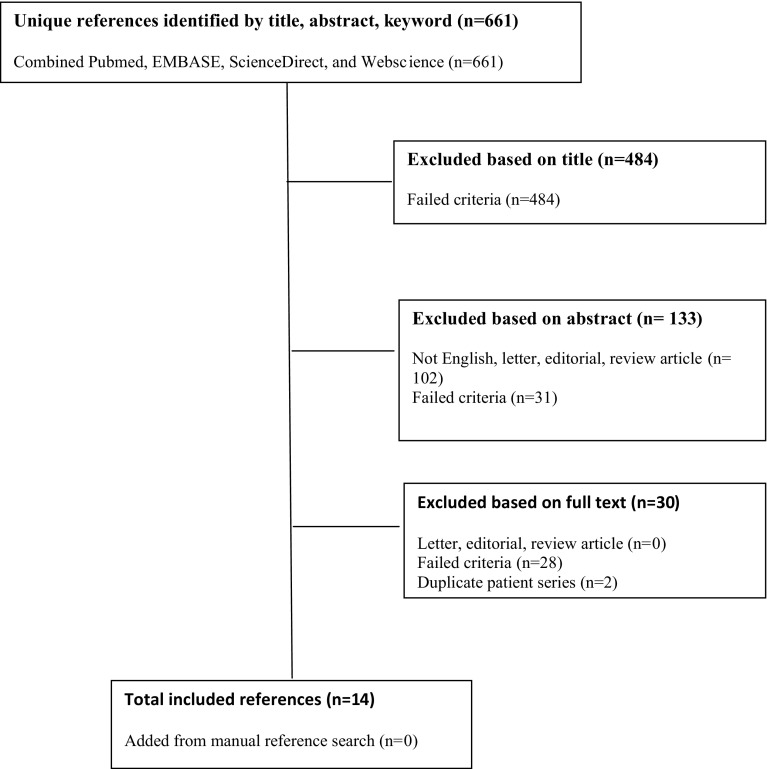
Flow diagram outlining the systematic review process used in this study

We identified 661 initial manuscripts using our search terms (Fig. 1). Four hundred and eighty-four articles were excluded following review of the article title because of irrelevance to the study question. One hundred and thirty-three were then excluded following a review of the abstract. Of these, 102 were either not written in English or were review articles or case reports, and 31 failed the above study criteria. This left 44 articles which required a full-text review. Of these, 30 were excluded following a full-text review because they did not meet our study inclusion criteria. In particular, two studies reported on treatment for both acute and subacute fractures (>6 weeks from injury) [12, 13]. One of these did not provide outcomes data specifically for acute fractures and was excluded [12]. In the other study, outcomes for acute fractures could be determined, and it was included [13]. Two sets of two articles [14–17] within the 44 full-text-reviewed papers were from the same group of authors reporting on the same or similar cohorts of patients at a later time point. In both cases, the more recent article was included [15, 17].

Three studies additionally reported on outcomes following IMN of fracture dislocations [18–20], and three studies additionally reported on outcomes following IMN of combined proximal humerus and shaft fractures [11, 21, 22]. Only two of these studies were included, as explicit outcomes data for acute proximal humerus fractures could be elicited [11, 19]. Finally, one study reported on acute fractures, fracture dislocations, and combined proximal humerus and shaft fractures [23]. Demographic and outcomes data were extracted for the acute fracture subgroup only. A total of 14 articles were ultimately included [7, 8, 11, 13, 15, 17, 19, 23–29].

Functional outcomes were measured using a variety of scoring systems: American Shoulder and Elbow Surgeons (ASES) [8, 27, 29], Constant score (CS) [2, 7, 8, 17, 26, 29], modified Constant score (mCS) [7, 17], relative Constant score (rCS) [19, 20, 26], Neer score [11, 13, 23, 24, 27], relative Neer score (rNeer) [26], Simple Shoulder Test (SST) [8], Oxford Shoulder Score (OSS) [17], Japanese Orthopaedic Association shoulder score (JOA) [25], and Shoulder Pain and Disability Index (SPADI) [28]. The JOA score is categorized as excellent (85–95 points), satisfactory (75–84 points), unsatisfactory (65–74 points), and poor (<65 points) [25]. The SPADI score is categorized as excellent (0–25), good (26–50), fair (51–75), and poor (76–100) [28]. Relative scores refer to the comparison of the operative shoulder with the contralateral, noninjured shoulder, expressed as a percentage.

Ten of the studies reported the use of statistical analysis [7, 8, 15, 17, 19, 23, 24, 26, 27, 29]. Each of the selected studies contributed data to the patient demographics. In situations where more than one study provided data for any of the outcome measures, the data were pooled and frequency-weighted means were calculated. The frequency-weighted mean represents the mean from each individual study weighted by the number of patients in that study. A standard Student *t* test was used to compare the frequency-weighted means for the demographic and outcomes data. *p*-Value <0.05 was considered statistically significant.

## Results

Nine studies were published as level IV evidence [7, 8, 11, 13, 23, 28], three as level III evidence [17, 19, 27], and one each as level II [15] and level I evidence [29]. Three studies included a comparative operative group using a locked proximal humeral plate [15, 17, 29]. No study included a nonoperative control group. The patients were operated on between 1993 and 2007, although one study did not provide this information [28]. All of the studies utilized either inclusion or exclusion criteria. Four studies were performed across multiple institutions [7, 15, 19, 26]. Nine studies reported on the number of surgeons involved, with a mean of 2.4 amongst those studies [7, 8, 11, 17, 23, 26–29].

### Demographics

Demographic data are provided in Table 1. There were a total of 529–563 patients in the 14 studies at baseline. The final patient total was 448. Using the Neer classification, 185 (41 %) had two-part fractures, 230 (51 %) had three-part fractures, and 33 (13 %) had four-part fractures. The frequency-weighted mean age was 64.3 years. All studies except for one reported on patient gender [11], with 338 females (71 %) and 139 males (29 %). Two studies provided only baseline age and gender demographics; therefore, the gender tally adds up to more than the final study patient number [15, 19]. Only four studies reported the dominance of the operated extremity [8, 15, 28, 29], with 50 % involving the dominant shoulder. The frequency-weighted mean follow-up was 22.6 months (range 12–65 months), and the mean minimum follow-up was 16.4 months. Six studies explicitly reported on the mean time from injury to surgery, with a frequency-weighted mean of 4.9 days (range 1.1–9.4 days) [13, 23, 25–27, 29].

**Table 1:** Demographic and operative details of included studies

Authors	Publication date	Type of study	No. of patients		Neer fracture type			Mean age (years)	Female/male	Mean follow-up (months)	Minimum follow-up (months)
			Baseline	Final	Two-part	Three-part	Four-part				
Hatzidakis et al. [7]	2011	Retrospective	48	38	38	0	0	65	28/10	20	12
Nolan et al. [8]	2011	Retrospective	18	13	9	4	0	71	10/3	42	24
Adedapo et al. [11]	2001	Retrospective	16	16	0	10	6	67.1	NR	12	12
Lin et al. [13]	1998	Retrospective	16–18	16	6	0	0	67.6	10/6	19.6	14
Konrad et al. [15]	2012	Prospective	58^a^	47	0	47	0	64.8	447/11	12	12
Trepat et al. [17]	2012	Retrospective	15	13	3	0	0	64.5	7/6	12	12
Gradl et al. [19]	2007	Prospective	69–96^b^	69	7	35	17	67.2	67/36	12	12
Lin et al. [23]	2006	Prospective	22–27	22	0	22	0	53.3	10/12	23.9	19
Kazakos et al. [24]	2007	Retrospective	31	27	16	11	0	65.9	17/10	12	12
Koike et al. [25]	2008	Retrospective	54	54	29	22	3	66	44/10	18	13
Linhart et al. [26]	2007	Retrospective	97	51	5	31	5	68.4	39/12	12	12
Park et al. [27]	2012	Retrospective	43	43	0	43	0	60.2	34/9	65	35
Sforzo et al. [28]	2009	Retrospective	14	14	7	5	2	56	9/5	40	12
Zhu et al. [29]	2011	Prospective	28	25	5	0	0	54.8	16/9	36	36
Totals			529–563	448	185	230	33		338/139^c^		
Frequency-weighted mean								64.3		22.6	16.4

NR, not reported

^a^Only baseline demographic data provided

^b^Four four-part fracture dislocations and 12 fractures with shaft extension excluded. Mean age and follow-up reported for entire baseline cohort of 112 patients, but weighted for 69 patients

^c^Total does not add up to total patient number given (b)

### Surgical technique

Nine studies reported performing the surgery in the beach-chair position [8, 13, 17, 19, 23–26, 28], one used the lateral position [15], two used the supine position [11, 29], and one used either the beach-chair or lazy lateral position [7]. One study did not report patient positioning [27]. While all studies (406 patients) utilized a deltoid-splitting approach, one predominately used the deltopectoral approach (42 patients) [27]. The most commonly used intramedullary device was the Polarus nail (Acumed, Beaverton, OR, USA) (36 %). Other intramedullary nails included the Targon PH nail (Aesculap, Tuttlingen, Germany) (27 %), Synthes PHN (Synthes GmbH, Oberdorf, Switzerland) (16 %), Stryker T2 nail (Stryker Orthopaedics, Mahwah, NJ, USA) (9 %), Humeral Locked Nail (United, Taipei, Taiwan) (8 %), Synthes EX spiral blade (Synthes, West Chester, PA, USA) (2 %), and the Uniflex humeral nail (Biomet, Warsaw, IN, USA) (1 %).

### Functional outcomes

Functional outcomes are reported in Table 2. Three studies (*n* = 81) reported ASES scores, with a frequency-weighted mean of 84.3 [8, 27, 29]. Seven studies (*n* = 225) reported the CS, with a frequency-weighted mean of 72.8 [7, 8, 11, 17, 19, 26, 29]. The rCS was reported in three studies (*n* = 167), with a frequency-weighted mean of 81.4 [15, 19, 26]. Two studies (*n* = 51) reported the mCS, with a frequency-weighted mean of 91.9 [7, 17]. Five studies (*n* = 124) reported a Neer score, with a frequency-weighted mean of 84.5 [11, 13, 23, 24, 27]. One study (*n* = 51) reported a rNeer score of 84.7 [26]. One study (*n* = 13) reported a mean OSS score of 19.8 [17]. One study (*n* = 54) reported a mean JOA score of 81.0 [25]. One study (*n* = 13) reported a mean SST score of 6.8 [8]. One study (*n* = 14) reported a mean SPADI score of 30 [28].

**Table 2:** Postoperative outcomes

Authors	Postoperative scores (mean)					Pain scores								Range of motion				
	ASES	CS	rCS	mCS	Neer	CS (0–15)	JOA (0–30)	VAS	Neer (0–35)	None	Mild	Mod	Severe	FE (°)	Abd (°)	Ext (°)	ER (°)	IR (°)
Hatzidakis et al. [7]	—	71.0	—	97.0	—	13.0	—	—	—	—	—	—	—	132.0	—	—	—	—
Nolan et al. [8]	67.8	60.5	—	—	—	—	—	2.3	—	—	—	—	—	120	—	—	38.8	T4 (1), T9 (2), T10 (1), T12 (1), L1 (2), L2 (2), L4 (3), L5 (1)
Adedapo et al. [11]	—	80.4	—	—	75.7	—	—	—	—	8.0	5.0	1.0	2.0	143.8	141.9	—	48.8	75.0
Lin et al. [13]	—	—	—	—	87.2	—	—	—	—	—	—	—	—	153.8	154.7	36.3	48.1	65.3
Konrad et al. [15]	—	—	89.0	—	—	—	—	—	—	—	—	—	—	—	—	—	—	—
Trepat et al. [17]	—	63.8	—	76.9	—	—	—	—	—	8	3	1	1	131	121.5	—	38.8	—
Gradl et al. [19]	—	69.9	75.6	—	—	—	—	—	—	—	—	—	—	—	—	—	—	—
Lin et al. [23]	—	—	—	—	85.3	—	—	—	32.7	—	—	—	—	137.3	133.9	32	39.3	56.6
Kazakos et al. [24]	—	—	—	—	83.7	—	—	—	—	—	—	—	—	—	—	—	—	—
Koike et al. [25]	—	—	—	—	—	—	26	—	—	—	—	—	—	—	—	—	—	—
Linhart et al. [26]	—	71.2	82.1	—	—	—	—	—	—	—	—	—	—	—	—	—	—	—
Park et al. [27]	86.0	—	—	—	87	—	—	—	—	—	—	—	—	—	—	—	—	—
Sforzo et al. [28]	—	—	—	—	—	—	—	—	—	—	—	—	—	105.6	—	—	36.5	—
Zhu et al. [29]	90.0	93.3	—	—	—	—	—	0	—	—	—	—	—	160.8	—	—	47.8	T8
Total (no. of patients)										16.0	8.0	2.0	3.0					
Frequency-weighted mean	84.3	72.8	81.4	91.9	84.5	13.0	26.0	0.8	32.7					137.3	138.4	33.8	43.1	
Standard deviation	7.4	8.2	5.5	8.8	3.7									15.6	11.3	2.1	4.9	

*ASES* American Shoulder and Elbow Surgeons, *CS* Constant score, *rCS* relative Constant score, *mCS* modified Constant score, *JOA* Japanese Orthopaedic Association, *VAS* visual analogue scale, *FE* forward elevation, *Abd* abduction, *Ext* extension, *ER* external rotation, *IR* internal rotation

### Pain outcomes

Pain scores were reported as a component of either the CS [7] or the Neer score [23], visual analogue scale (VAS) [8, 29], or subjectively as mild, moderate or severe [11, 17]. One study (*n* = 38) reported a mean Constant pain score of 13.0 [7]. This score is given on a scale of 0–15, where a score of 15 represents no pain. One study (*n* = 22) reported a mean Neer pain score of 32.7 on a scale of 0–35, where a score of 35 represents no pain [23]. Two studies reported a mean VAS score using a scale of 0–10, with a frequency-weighted mean of 0.8 [8, 29]. Two studies (*n* = 29) reported pain as either mild, moderate or severe [11, 17]. Sixteen patients reported no pain, eight had mild pain, two had moderate pain, and three had severe pain.

### Range of motion

Range-of-motion outcomes were reported in eight studies [7, 8, 11, 13, 17, 23, 28, 29]. These included active forward elevation [7, 8, 11, 13, 17, 23, 28, 29], abduction [11, 13, 17, 23], external rotation at the side [8, 11, 13, 17, 23, 28, 29], extension [13, 23], and hand-in-back internal rotation [8, 11, 13, 23, 29]. Eight studies (*n* = 141) reported a frequency-weighted mean forward elevation of 137.3°. Four studies (*n* = 54) reported a frequency-weighted mean active abduction of 138.4°. Two studies (*n* = 38) reported a mean extension of 33.8°. Seven studies (*n* = 119) reported active external rotation with a frequency-weighted mean of 43.1°. Three studies (*n* = 54) evaluated internal rotation based on arc of motion with a frequency-weighted mean of 64.6° [11, 13, 23], whereas two (*n* = 38) reported values for maximum hand-in-back internal rotation, with a range of T2 through buttock [8, 29].

### Complications

All studies reported both radiographic outcomes and complications following IMN of acute, displaced proximal humerus fractures. The overall radiographic healing rate in this series was 99.3 % (445/448). Only five studies explicitly reported radiographic parameters for fracture malunion [8, 17, 25, 27, 28], four studies explicitly reported a definition of nonunion [8, 17, 25, 28], and eight studies formally measured the final radiographic neck–shaft angle (NSA) [7, 8, 15, 17, 23, 25, 27, 28]. Five studies formally reported the loss of NSA during the postoperative period [7, 8, 17, 27], and one study only reported patients with >10° loss of NSA [29]. One study (*n* = 16) reported on complications of a patient group which included proximal humeral fractures with shaft extension [11]. Furthermore, one study (*n* = 47) included patients with <12 months follow-up in their report on complications [15]. These patients were thus excluded from analysis of the complications for acute proximal humeral fractures, leaving a total of 385 patients.

**Fig. 2:**
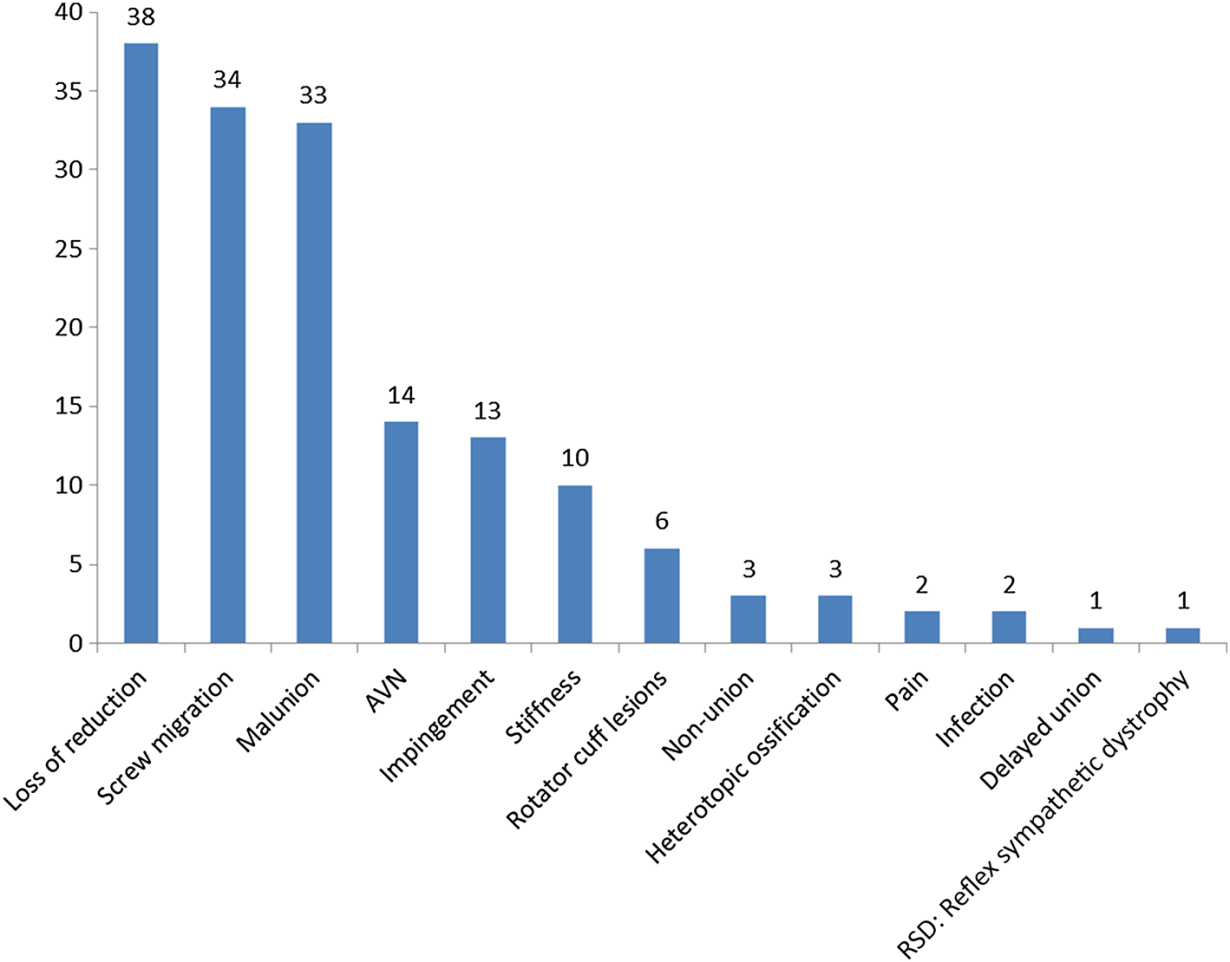
Summary of complications

There were 61 (15.8 %) secondary surgeries, mostly for removal of migrated proximal screws. There were 160 (41.5 %) reported complications, with 38 instances of secondary loss of reduction (10 %), 34 instances of screw migration or perforation into the joint (9 %), 33 instances of malunion (9 %), 14 instances of avascular necrosis (4 %), and 13 instances of subacromial impingement (4 %). The remaining complications are depicted in Fig. 2. No case of nerve or vascular injury was reported in this patient series.

### Outcomes and complications by fracture pattern

Further analysis was performed to stratify the functional outcomes and complications by fracture pattern. The results are given in Table 3. The frequency-weighted ASES score for two-part fractures was found not to be statistically different from the score for three-part fractures [85.5 versus 83.5, *p* = 0.6, 95 % CI (−4.8, 8.8)]. The frequency-weighted mean CS for two- and three-part fractures were statistically greater than for four-part fractures [*p* = 0.007, 95 % CI (2.9, 18.5) and *p* = 0.0009, 95 % CI (5.0, 18.8), respectively], but were not different from each other. The frequency-weighted mean forward elevation for two- and three-part fractures were greater than for four-part fractures [*p* < 0.0001, 95 % CI (22.9, 65.9) and *p* = 0.001, 95 % CI (19.3,74.7), respectively], but were not statistically different from each other [*p* = 0.7, 95 % CI (−14.4, 9.4)]. The frequency-weighted mean abduction was greater amongst two-part fractures versus four-part fractures; however, no significant differences were found in abduction and external rotation between three- and four-part fractures.

**Table 3:** Subgroup outcomes analysis

Outcome	Two-part fracture		Three-part fracture		Four-part fracture		*p*-Value		
	(mean ± SD)	*N*	(mean ± SD)	*N*	(mean ± SD)	*N*	Two versus three	Two versus four	Three versus four
Forward elevation	140.4 ± 29.2°	95	143.0 ± 35.8°	38	96.0 ± 33.1°	8	0.7	<0.0001	0.001
Abduction	154.7 ± 30.6°	16	140.5 ± 31.8°	32	120.0 ± 20.0°	6	0.2	0.04	0.1
External rotation	45.4 ± 17.7°	57	44.0 ± 16.7°	38	33.8 ± 18.3°	8	0.7	0.1	0.09
Constant score	74.3 ± 17.7	104	75.5 ± 15.6	127	63.6 ± 19.9	27	0.6	0.007	0.0009
Neer score	87.2 ± 7.7	16	85.3 ± 9.9	122	62.5 ± 11.5	6	0.5	0.0001	0.0001
ASES score	85.5 ± 17.2	34	83.5 ± 13.5	47	na	na	0.6	na	na

*ASES* American Shoulder and Elbow Surgeons, *na* not applicable

Complication data and reoperation rates for seven studies (*n* = 125) involving two-part fractures [7, 8, 13, 17, 19, 28, 29] and for five studies (*n* = 109) for three-part fractures [8, 19, 23, 27, 28] were analyzed. Only two studies (*n* = 19) explicitly reported complications for four-part fractures [19, 28]. The complication rate for two-part fractures was 33.6 %, and the reoperation rate was 13.6 %. For three-part fractures, the complication rate was 57.8 % with a reoperation rate of 17.4 %. There was no significant difference in reoperation rate (*p* = 0.5) between two- and three-part fractures, though two-part fractures had a significantly lower complication rate [*p* = 0.0002, 95 % CI (11, 36 %)]. In both instances, secondary fracture displacement/fracture malunion accounted for the majority of complications, followed by screw migration/glenohumeral joint penetration. Despite the small number of four-part fractures for which complications could be assessed, a reoperation rate of 63.2 % was found. The reoperation rate for both two- and three-part fractures was significantly less than for four-part fractures [*p* < 0.0002, 95 % CI (22, 64 %) and (26, 68 %), respectively]. There were 29 reported complications amongst the four-part fracture cohort.

## Discussion

Proximal humerus fractures remain common amongst the elderly, behind fractures of the hip and distal radius [4]. Complex three- and four-part fractures account for >50 % of cases in patients older than 60 years [3, 4]. Despite their prevalence, optimal treatment remains controversial. Zyto et al. found no functional difference between tension band fixation versus nonsurgical management of displaced three- and four-part proximal humerus fractures at 1 and 3–5 year follow-up [30]. Others have shown comparable clinical results for four-part fractures treated with hemiarthroplasty or nonoperatively [31]. Reverse shoulder arthroplasty may play a role in elderly patients with complex three- and four-part proximal humerus fractures [10, 32]. Percutaneous fixation has demonstrated good midterm results, although avascular necrosis (AVN) rates are high [5]. While locked proximal humerus plating has become popular [6, 33, 34], the complication and reoperation rates remain high [34]. As such, multiple operative techniques have been recommended for treatment of displaced fractures, including percutaneous fixation [5], ORIF [6, 34], IMN [7, 8], hemiarthroplasty [9], and RSA [10]. Intramedullary nailing has been reported to provide clinical outcomes comparable to locking plates [15, 19, 29], with less soft tissue dissection and a possibly improved complication profile.

In the current systematic review on the outcomes of IMN for displaced proximal humerus fractures, we found a frequency-weighted mean patient age of 64.3 years. The majority of patients were female, corresponding to the gender that most frequently sustains this fracture. The most common fracture pattern treated was three-part fractures, followed by two-part fractures. Few studies included four-part fractures, likely reflecting the technical difficulty in utilizing IMN for this pattern, in addition to the number of alternative implants available. A systematic review of locking plates for proximal humerus fractures similarly found that three-part fractures were most commonly treated, followed by two-part fractures [34]. Their mean age was 62 years, with a majority of patients being female. In comparison, patients undergoing RSA for displaced proximal humerus fractures tend to be older with the majority of cases involving four-part fractures [32]. In a comparative study of HA versus RSA, the mean age for patients undergoing HA was 74.1 and 74.8 years for RSA [35]. In younger, more active patients, operative fixation of displaced proximal humerus fractures has generally been favored over the use of shoulder arthroplasty.

We determined a frequency-weighted mean CS of 72.8 and ASES score of 84.3. The CS is comparable to the score of 73.6 reported in a systematic review of locked plating for proximal humerus fractures [34]. Additionally, the authors found the score for four-part fractures (67.7) to be statistically worse than for two-part fractures (77.4). The CS for two-part fractures (74.3) in the current study was statistically better than for four-part fractures (63.6), but it was not different from three-part fractures (75.5). Similar findings were made with respect to the Neer score, suggesting that the outcomes for two- and three-part fractures treated with IMN are comparable. Our overall frequency-weighted mean forward elevation, abduction, and external rotation were 137.3°, 138.4°, and 43.1°, respectively. Forward elevation for both two- and three-part fractures (140.4° and 143.0°, respectively) were significantly greater than for four-part fractures (96°), but no statistical difference was noted for abduction and external rotation for three- versus four-part fractures. Forward elevation and abduction reported by Sproul et al. were 98° and 103°, though based on only two studies [34]. Solberg et al. reported a higher CS following locked plating of three-part proximal humerus fractures compared with hemiarthroplasty, though this difference was not significant for four-part fractures [6]. Harrison and colleagues reported a mean ASES score of 82 at a minimum of 3 years following closed reduction percutaneous pinning of predominately three- and four-part fractures. The mean forward elevation was 140° and external rotation was 41°, though no analysis was performed by fracture pattern [5]. Our findings of fair clinical outcomes and ROM following IMN of four-part fractures emphasize the need for further study into the use of fracture fixation versus arthroplasty in this difficult patient group.

The most common complication associated with IMN in this review was secondary loss of reduction (24 %) followed by fracture malunion (21 %). Many complications occur beyond 12 months, and our mean follow-up was 22.6 months. Loss of reduction can be associated with proximal screw migration or screw penetration into the glenohumeral joint. Malunion has been associated with poor clinical outcomes [8, 18]. Despite its correlation with outcome, only five studies reported the loss of NSA and only eight of the studies reported a final NSA. Park et al. demonstrated the importance of restoring and supporting the medial calcar with a screw [27]. The authors also utilized tension band sutures and noted improved radiographic and clinical outcomes. The benefits of fracture augmentation using calcium sulfate cement and placement of inferomedial screws have been demonstrated with locking plates and may play a role with intramedullary nailing [36, 37]. In our study, the overall AVN incidence was 4 %, lower than that reported by Sproul et al. for locked plating (10.8 %) [34] and Harrison et al. for percutaneous pinning (26 %) [5]. This finding may be expected given the less invasive insertion of IMN, though it may not present radiographically for years [5]. Kloub and colleagues found that reduction quality influenced AVN development, with 2 % complete necrosis following excellent reduction compared with 60 % complete necrosis following poor reduction [20]. AVN was associated with worse clinical outcome. Perhaps not unexpectedly, complications amongst two-part fractures (33.6 %) were lower in our study than amongst three-part fractures (57.8 %). The majority of these complications were related to loss of reduction and malunion, similar to that reported for locked plating [6, 33, 34]. Future studies need to better stratify complications by injury pattern to better understand which fractures would benefit from the use of IMN. Furthermore, fracture augmentation strategies may potentially reduce postoperative loss of reduction.

Our overall reoperation rate of 15.8 % compares well with that reported for locked plating. Sproul and colleagues reported a reoperation rate of 13.8 %, mostly for screw penetration [34]. Most of the reoperations in our study were related to proximal screw migration. Proximal screw migration may be decreased through the use of an end-cap as well as threaded bushings within the nail to minimize screw back-out [7, 29]. The nail should be inserted at least 5 mm below the subchondral bone of the humeral head and proximal screw lengths fluoroscopically verified [8, 18]. A more medial articular entry point may cause less damage to the rotator cuff as the more medial aspect of rotator cuff has more vascularity [7, 8, 18], though rotator cuff symptoms can persist despite meticulous repair [8]. The reoperation rate for two-part and three-part fractures in our study was 13.6 and 17.4 %, respectively. As these result primarily from screw back-out and fracture displacement, meticulous surgical technique and newer implant designs may reduce their incidence. Given the high reoperation rate amongst four-part fractures (63.2 %), caution should be exercised in selecting IMN for this fracture pattern and preoperative discussions should be held with the patient regarding this risk.

There are several limitations of this systematic review, primarily related to the inherent limitations of the studies on which this review was based. There were 10 retrospective studies and 4 prospective studies with the majority (64 %) published as level IV evidence. Additionally, a variety of outcome measures and nonuniform assignment of complications to each individual patient were used. Future studies with standardized use of outcomes measures and strict definitions for complications are needed. Despite this, we were able to pool the data for the Constant, Neer, and ASES scores to generate frequency-weighted means. Two studies did not report demographic data for acute fractures only, and their age and gender distribution were weighted only for the cohort of interest. This may have affected the reported mean age, although the gender distribution was similar to the remaining studies. Complication data were often reported for the entire cohort of patients, including instances other than for acute fracture. We therefore excluded complication data unless explicitly reported for the acute fractures, and this may have affected the calculated complication profile following IMN. Finally, multiple intramedullary nail designs with differing proximal screw insertion configurations and the variable use of an endcap were included, which may have overestimated the complications. Our review was strengthened by the final patient number and the weighted mean follow-up of 22.6 months with a minimum of 16.4 months, which is adequate for a study involving fractures. Additionally, we were able to perform a subgroup analysis amongst the fracture patterns for ROM and functional outcomes. This study represents the first systematic review that the authors are aware of assessing the use of intramedullary nailing for acute proximal humerus fractures.

Intramedullary nailing of acute two- and three-part proximal humerus fractures yields satisfactory clinical and functional results. Only fair clinical and functional results were reported for four-part fractures, suggesting that further studies with larger patient numbers are needed to determine the role of intramedullary nailing for four-part fractures. Further, the complication profile and reoperation rate, in particular loss of reduction, remain high regardless of fracture pattern. Newer implant designs and use of augmentation techniques (i.e., rotator cuff sutures, tricalcium phosphate cement) may reduce its incidence.
